# A Novel Abutment Screw Head Design with an Integrated Retrieval Feature: An In Vitro Study

**DOI:** 10.3390/dj14090613

**Published:** 2026-09-21

**Authors:** Ricardo Batista, André Moreira, Nuno Alves, Maria Helena Figueiral, João Sampaio-Fernandes

**Affiliations:** 1 Department of Prosthodontics, Faculty of Dental Medicine, University of Porto, 4200-393 Porto, Portugal; andre.luis.sa.moreira@gmail.com (A.M.); hfigueiral@gmail.com (M.H.F.); sampaiofernandes@gmail.com (J.S.-F.); 2 Institute of Science and Innovation in Mechanical and Industrial Engineering (INEGI), 4200-465 Porto, Portugal; 3 Centre for Rapid and Sustainable Product Development, ESTG (School of Technology and Management), Polytechnic Institute of Leiria, 2411-901 Leiria, Portugal; nuno.alves@ipleiria.pt

**Keywords:** dental abutments, prosthetic screw, removal torque, dental restoration failure

## Abstract

**Background/Objectives**: This study aimed to present the design of a novel abutment screw head featuring an integrated retrieval mechanism and to evaluate the retrievability of four prototype designs. **Methods**: In this in vitro mechanical study, the modified screws were produced by replicating the macrogeometry of the Brånemark and Klockner VEGA abutment screws and incorporating a secondary screw chamber below the original one, with a 1.0 mm or 0.9 mm hexagonal design. Sixty screws were manufactured, fifteen for each group: BC0.9, BC1.0, KC0.9, and KC1.0. Retrieval success and the relationship between peak tightening and loosening torque were evaluated (α = 0.05). **Results**: All screws tolerated tightening at a torque 20% above the manufacturer’s recommendation. Most specimens from BC0.9 and KC0.9 failed during loosening, while all specimens in BC1.0 and KC1.0 were successfully retrieved. Regarding peak tightening torque and the loosening-to-tightening ratio, neither prototype exhibited a statistically significant correlation. BC1.0 demonstrated a statistically significant positive correlation between peak tightening torque and peak loosening torque, whereas KC1.0 showed a weaker relationship; detailed correlation coefficients and *p*-values are reported in the Results. **Conclusions**: Under the in vitro conditions tested, the prosthetic screw incorporating a 1.0 mm secondary retrieval head demonstrated reliable retrieval performance, supporting its potential as a simple retrieval system. These findings represent a proof-of-concept and require further mechanical and clinical investigation before clinical application.

## 1. Introduction

The long-term success of screw-retained implant-supported restorations depends on the mechanical stability of the implant–abutment complex, which is strongly influenced by the interface design and by the abutment screw itself [1,2,3]. The screw generates and maintains the preload that keeps the mating surfaces in intimate contact, and loss of preload is a primary factor behind mechanical complications such as screw loosening and fracture [1,2,4,5,6,7]. Conventional screw heads are optimised for torque transmission [8,9,10,11,12], but successive tightening and loosening, limited restorative space, cyclic loading, and non-axial forces can produce cumulative plastic deformation of the head, even at recommended torque values [9,13,14,15,16,17,18]. Contamination or the use of incompatible screwdrivers may aggravate this damage [15,16], and Ghaffari et al. observed a progressive increase in head surface area with repeated tightening, dependent on head geometry [9,16,18,19].

Once deformation exceeds the elastic range, torque transmission and retightening become unreliable, preload is progressively lost, and the screw may ultimately fracture, a complication associated with considerable clinical difficulty and risk of implant damage during retrieval [4,6,10,11,13,14,15,20,21,22,23,24]. When a compromised screw must be removed, clinicians rely on a range of retrieval techniques, from conservative options (manufacturer-specific extraction kits, elastomer-assisted engagement, or manual removal of loose fragments) [5,7,11,12,22,25] to intermediate mechanical methods (grinding, slot creation, or reverse-threaded extractors) [23,26,27,28], ultrasonic disengagement [29], commercial repair kits [30,31] and, ultimately, salvage approaches such as custom-cast restorations, retapping, or shortened screws [25,30,31,32,33,34].

A common limitation of all these approaches is that they become necessary only after the primary screwdriver interface is already damaged, and most rely on grinding, drilling, or dedicated instruments that may harm the implant connection; the constraints of limited restorative space further discourage modifications to the original screw [35,36]. No currently available solution allows a screw with a deformed primary head to be loosened directly before destructive measures are required.

To address this gap, the present in vitro study aimed to design and test the retrievability of four prototype abutment screws incorporating a secondary, independent retrieval interface (a lower screw chamber for a retrieval screwdriver) intended for use when the original chamber becomes damaged. It was hypothesised that, even when the screw was tightened at 20% above the manufacturer’s recommended torque, the secondary interface would still allow loosening and that its dimension (0.9 mm vs. 1.0 mm) would influence retrieval success.

## 2. Materials and Methods

This was an in vitro mechanical study. The primary outcome was retrieval success or failure of each screw through the secondary interface; secondary outcomes were the peak tightening torque, the peak loosening torque, and the proportional torque loss. Taking into consideration our previous study and the clinical limitations of implant rehabilitation, we developed a novel abutment screw design to address the issue of removing screws with deformed heads [13]. It was tested two types of implant connections: external and internal. For the external connection, the Brånemark 4.1 system (Nobel Biocare, Gothenburg, Sweden) was selected because it is one of the most widely used and extensively studied systems. For the internal connection, the Klockner VEGA RV system (Klockner Implant System, Escaldes-Engordany, Andorra) with Torx geometry (star-shaped with eight rounded lobes and excellent torque transmission characteristics) was selected.

**Figure 1:**
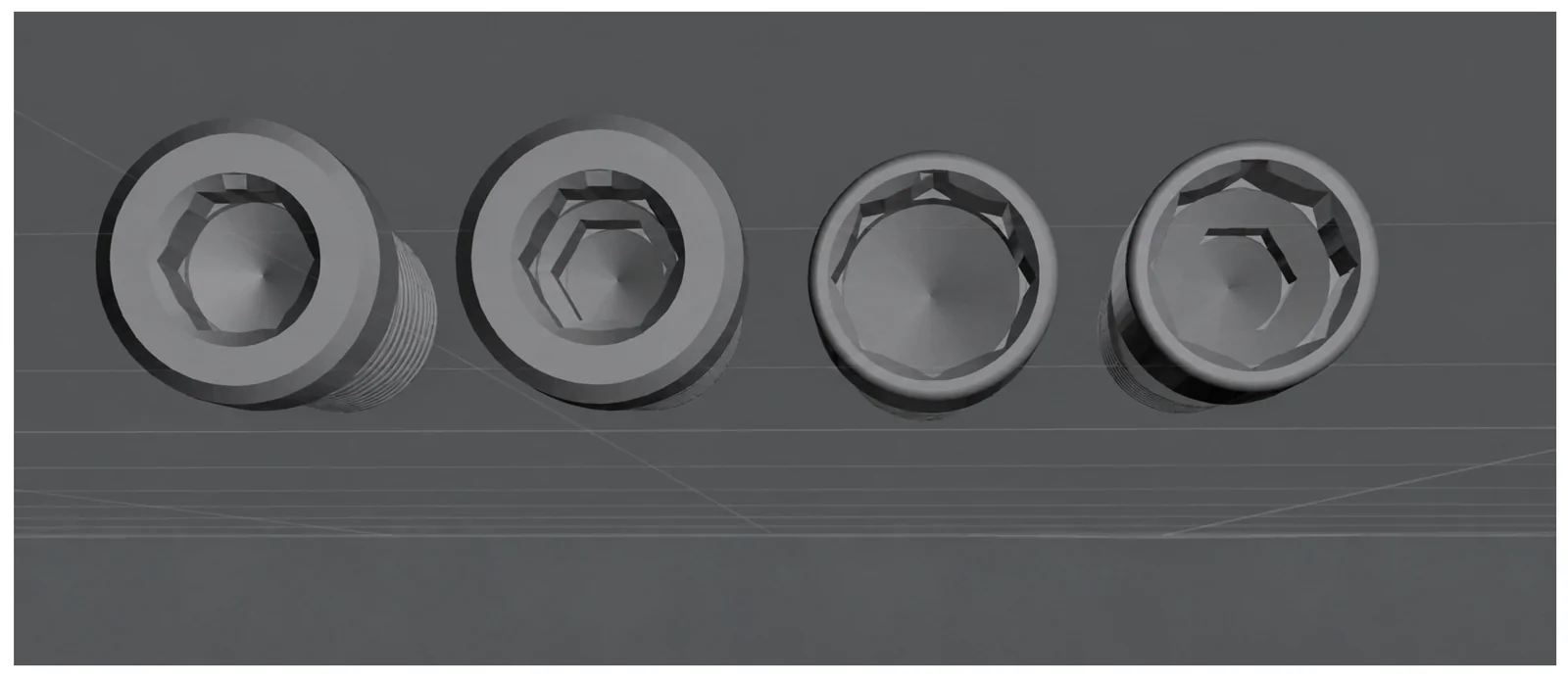
Upper view of screw head section. From right to left: commercial Brånemark screw, modified Brånemark-compatible screw with two engaging sections, commercial Klockner VEGA RV screw, and modified Klockner-compatible screw with two engaging sections.

**Figure 2:**
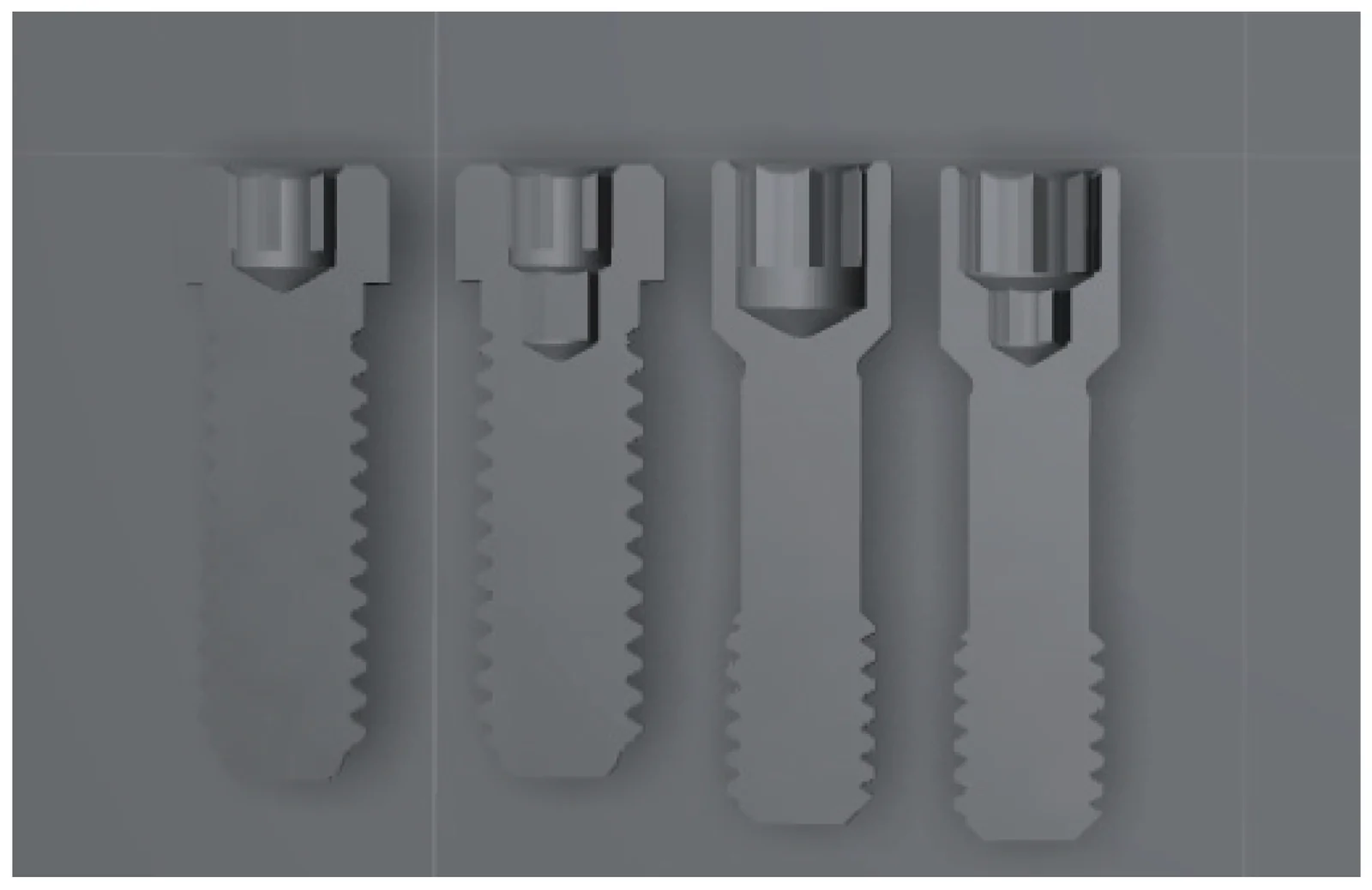
Longitudinal cross-sectional view, from left to right: commercial Brånemark hexagonal 1.2 mm screw, modified Brånemark-compatible screw, commercial Klockner VEGA RV, and modified Klockner-compatible screw.

The proposed screw head design consists of a secondary hexagonal interface incorporated into the lower portion of the original screw head (Figure 1 and Figure 2), which was manufactured by Osteotech (Osteotech, Tecnologia e Desenvolvimento Lda., Loures, Portugal). These modifications were applied to replicas based on the macrogeometry of Brånemark external screws and Klockner VEGA RV screws: Brånemark-compatible and Klockner-compatible screws, respectively.

**Figure 3:**
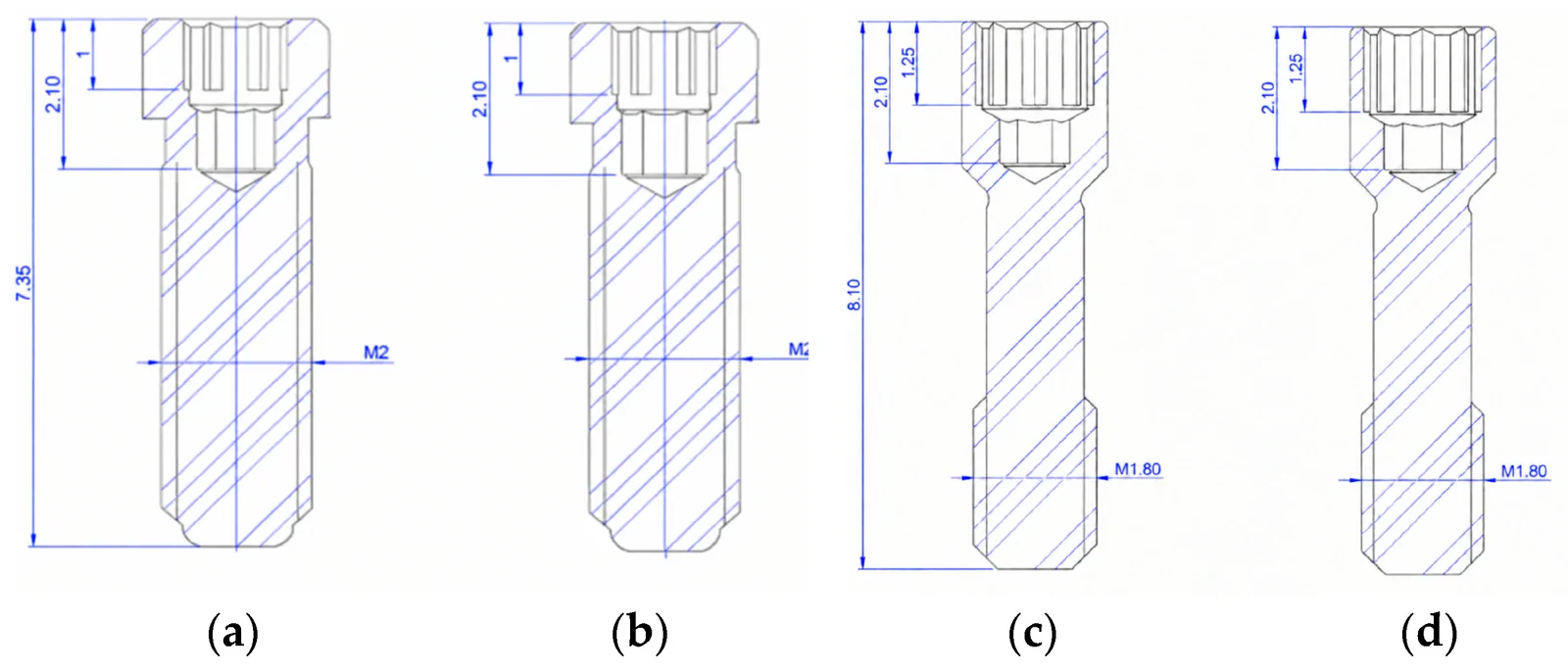
Longitudinal cross-section design of tested screws. (**a**) Modified Brånemark-compatible screw with 0.9 mm retrieval section; (**b**) Modified Brånemark-compatible screw with 1 mm hexagonal retrieval section. (**c**) Modified Klockner-compatible screw with 0.9 mm lower section; (**d**) Modified Klockner-compatible screw with 1 mm hexagonal lower section.

Interfaces with hexagonal head sections measuring 0.9 mm and 1.0 mm were tested. This design preserved compatibility with the original manufacturer’s screwdriver while incorporating an additional, smaller hexagonal interface (0.9 mm or 1.0 mm) intended for retrieval purposes and to evaluate which geometric configuration was better suited for this function. The original screw head height was maintained in the 0.9 mm Brånemark-compatible screws. In the other three types of screws, the vertical dimension of the screw head was increased, as illustrated in Figure 3. The total height of the double-geometry screw head was standardised at 2.1 mm for all groups.

**Figure 4:**
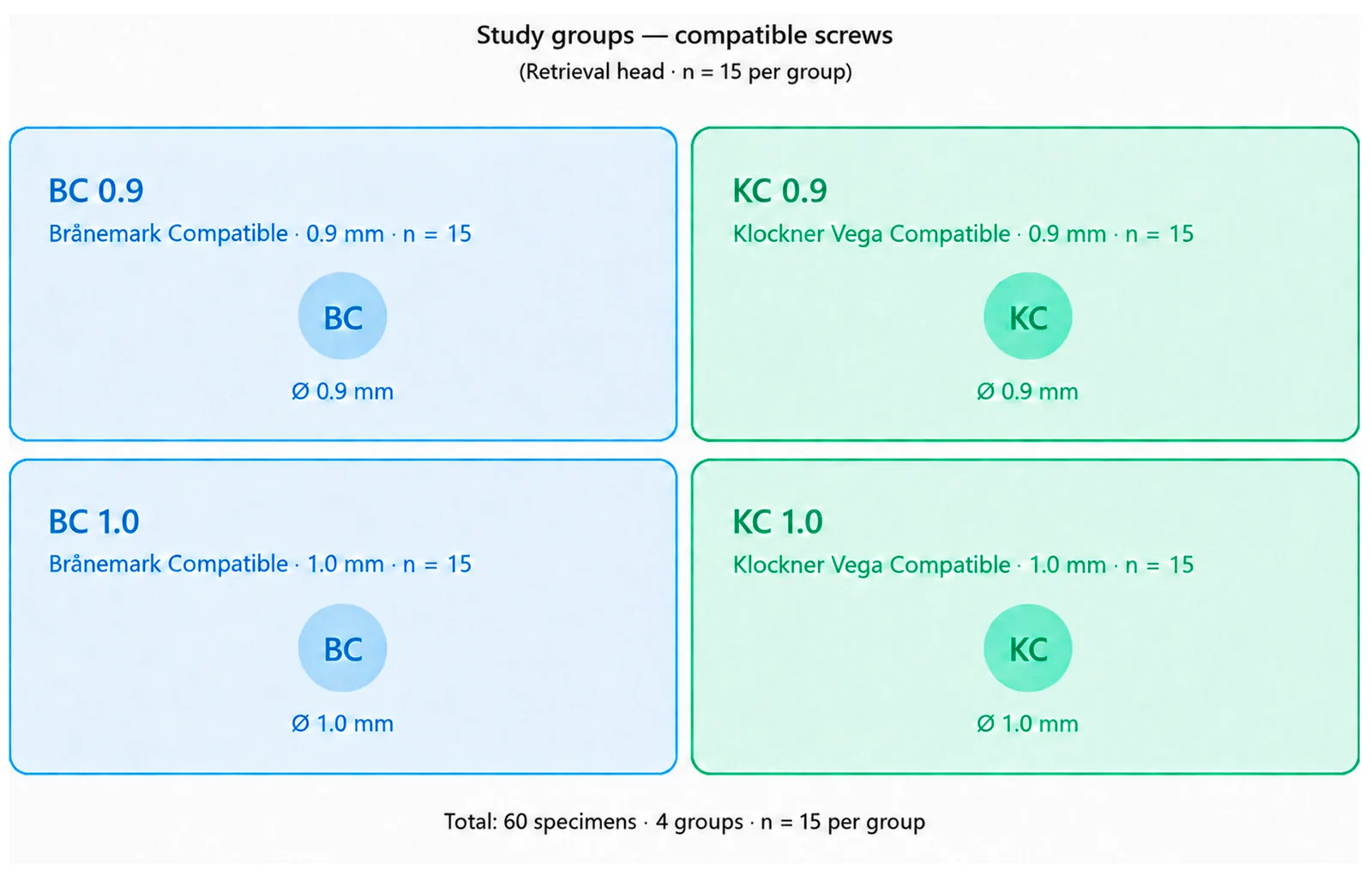
Study groups.

Four groups of fifteen specimens were created: BC0.9, BC1.0, KC0.9, and KC1.0. BC0.9 consisted of Brånemark-compatible screws (hexagonal 1.2 mm) with a secondary lower compartment with a 0.9 mm hexagonal section head for the retriever screwdriver. BC1.0 consisted of Brånemark-compatible screws (hexagonal 1.2 mm) with a secondary lower compartment with a 1.0 mm hexagonal section head for the retriever screwdriver. KC0.9 consisted of Klockner-compatible screws (star shape) with a secondary lower compartment with a 0.9 mm hexagonal section head for the retriever screwdriver. Finally, KC1.0 included Klockner-compatible screws (star shape) with a secondary lower compartment with a 1.0 mm hexagonal section head for the retriever screwdriver. Figure 4 shows the groups tested, indicating the brand of the screw’s macrogeometry, modifications, and the number of specimens.

**Figure 5:**
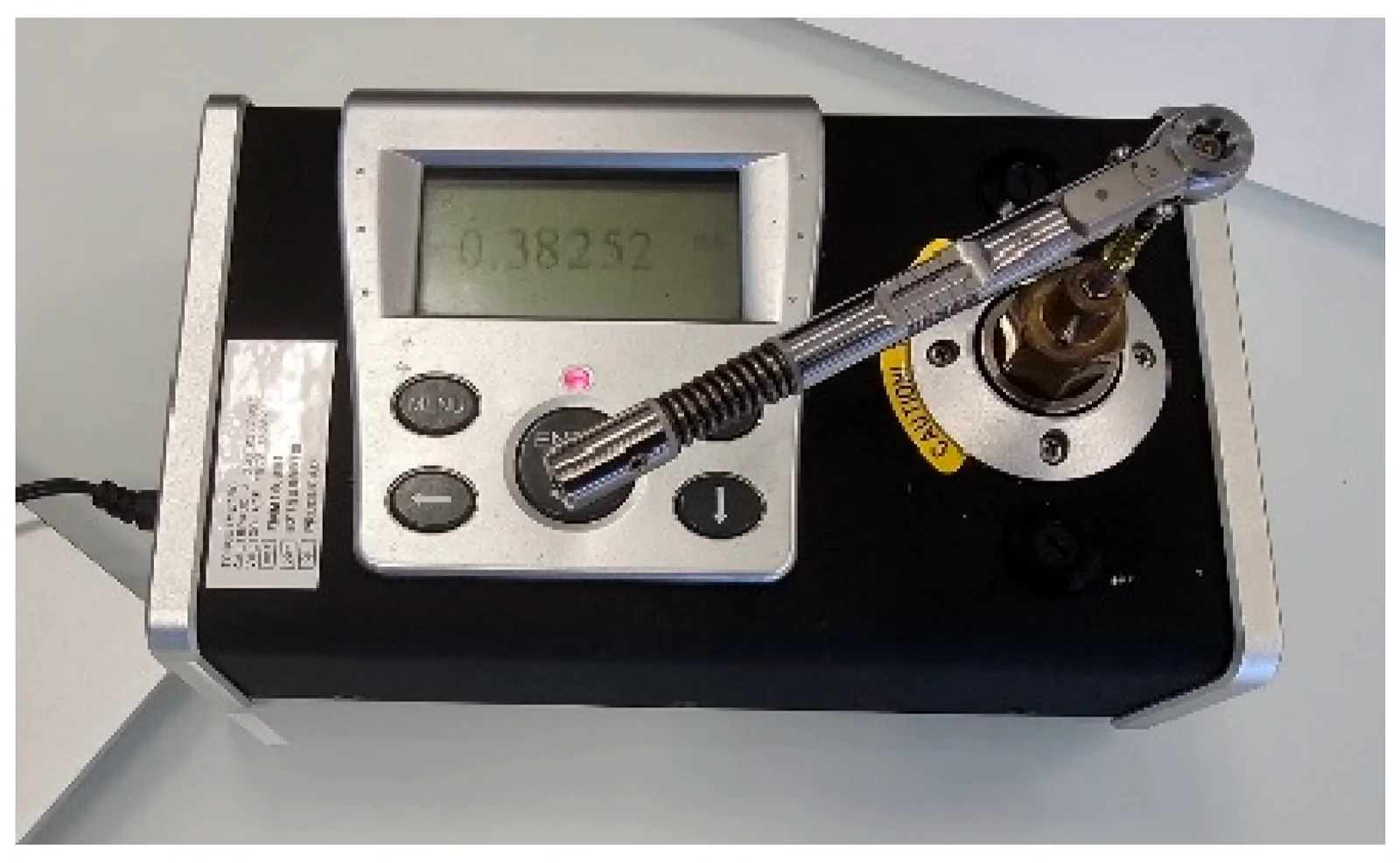
Torque measurement setup, showing the implant–abutment assembly mounted on the Mountz EZ-TorQ II digital torque analyser used to register peak tightening torque (through the primary head) and reverse loosening torque (through the secondary retrieval head).

The retrievability of the four different prototype designs was evaluated through the following standardised procedure. Each screw was first tightened through the primary head, using each brand’s original screwdriver, at the target torque (20% above the manufacturer’s recommendation) and left undisturbed for 10 min at room temperature under dry conditions to allow preload relaxation. The screw was then re-tightened through the primary head at the same target torque, and the peak tightening torque was recorded at this stage. After a second 10 min interval under the same conditions, the screw was loosened exclusively through the secondary retrieval head (0.9 mm or 1.0 mm interface), and the loosening (retrieval) torque was recorded. A new specimen was used for each screw, and no specimen was reused. All procedures were performed by a single trained operator under standardised axial alignment. Each screw was calibrated using a thread plug gauge (M2 Go/No-Go, Ref. DMM07.16, Serial No. 56102) and a thread ring gauge (M2 Go, Ref. DMM08.17, Serial No. 71035; M2 No-Go, Ref. DMM08.18, Serial No. 71036) (Messwelk GmbH, Kleinostheim, Germany) for individual validation. Torque was applied and measured using a Mountz EZ-TorQ II digital torque analyser (Mountz, Inc., San Jose, CA, USA; supplied by Hoffmann Group, Munich, Germany; Ref. EMM10.001; Lot EZ12080019; property of Osteotech), calibrated on 20 March 2024 and valid until 19 March 2027; peak tightening and reverse (loosening) torque values were registered directly by the instrument. The applied torque was set at 20% above the manufacturer’s recommendation: 40 Ncm for BC0.9 and BC1.0; 38 Ncm for KC0.9 and KC1.0. This protocol was adopted from our previous study [13] to reproduce a demanding, worst-case mechanical scenario consistent with reported clinical situations of over-tightening and cumulative loading, thereby providing a conservative test of the secondary retrieval interface and ensuring methodological continuity with our earlier work. Peak tightening torque (primary head) and reverse loosening torque (secondary head) values were measured for each screw (Figure 5). During loosening with the retrieval screwdriver, the screwdriver engagement angles were evaluated after each test.

To evaluate the mechanical relationship between tightening and loosening torque, two correlation analyses were performed for each group: (1) peak tightening torque vs. loosening-to-tightening ratio (%) and (2) peak tightening torque vs. peak loosening torque.

Fifteen specimens (n = 15) were tested per prototype. A formal a priori sample-size calculation was performed at the design stage using G*Power (version 3.1.9.7; Heinrich-Heine-Universität Düsseldorf, Düsseldorf, Germany). As the primary factor under investigation was the dimension of the secondary retrieval interface (0.9 mm versus 1.0 mm), the calculation was based on the comparison of two independent proportions corresponding to the anticipated retrieval success rates of these two s (50% for the 0.9 mm interface and 95% for the 1.0 mm interface), with a power of 80% and a significance level of α = 0.05, which yielded 15 specimens per group. The four experimental groups (BC0.9, BC1.0, KC0.9, and KC1.0) resulted from crossing this interface-dimension factor with the two implant–abutment connection types under study for external (Brånemark-compatible) and internal (Klockner-compatible) connections, so that the retrieval concept could be assessed in both connection designs, with each of the four groups comprising 15 specimens. Retrieval success (the primary, categorical outcome) was compared between the 0.9 mm and 1.0 mm secondary interfaces using Fisher’s exact test (two-tailed, α = 0.05), which is appropriate for proportions and for contingency tables containing small or zero cell counts. In another perspective, a sensitivity analysis indicated that, with n = 15 and α = 0.05 (two-tailed), the study had 80% power to detect a Pearson correlation of r ≥ 0.67 and 90% power to detect r ≥ 0.73. The correlation analyses were used to characterise the within-group mechanical relationship between tightening and loosening torque (i.e., the predictability of loosening from tightening), which is directly relevant to the reliability of a retrieval system; formal between-group inferential comparison was not the objective and was not feasible for the 0.9 mm groups, in which most specimens failed retrieval and no loosening measurements could be obtained. Both Pearson’s product-moment correlation coefficient (r) and Spearman’s rank correlation coefficient (ρ) were computed, with statistical significance assessed using the t-distribution (two-tailed, α = 0.05, df = 13).

## 3. Results

All screws from the four groups tolerated tightening at a torque value 20% above the manufacturer’s recommendation. In BC0.9, 13 screws could not be retrieved after tightening because of complete damage to the screwdriver engagement angles, whereas in KC0.9, five screws were also non-retrievable (Table A1). In contrast, in both BC1.0 and KC1.0, all fifteen screws were successfully unscrewed and retrieved. The number and percentage of successfully retrieved screws per group were as follows: BC0.9, 2/15 (13.3%); KC0.9, 10/15 (66.7%); BC1.0, 15/15 (100%); and KC1.0, 15/15 (100%). Retrieval success was significantly higher for the 1.0 mm interface than for the 0.9 mm interface. Pooling both implant systems, the 1.0 mm interface achieved 100% retrieval (30/30) versus 40% (12/30) for the 0.9 mm interface (Fisher’s exact test, *p* < 0.001). This difference remained significant within each system: Brånemark, 15/15 versus 2/15 (*p* < 0.001); Klockner, 15/15 versus 10/15 (*p* = 0.042). During testing of KC1.0, replacement of the 1.0 mm screwdriver became necessary after the third screw because the engagement angles were damaged, which could have compromised subsequent tests. Table 1 presents the bivariate correlation analysis, using both Pearson’s r and Spearman’s ρ to evaluate parametric and non-parametric estimates simultaneously for BC1.0 and KC1.0. Descriptive statistics for BC0.9 and KC0.9 were not calculated because most specimens failed during the loosening procedure, so valid loosening-torque measurements could not be obtained for these two groups (Table A1).

The mean peak tightening torque values were 38.79 Ncm for BC0.9, 48.17 Ncm for BC1.0, 38.47 Ncm for KC0.9, and 45.56 Ncm for KC1.0. When the retrieval screwdriver was used on the secondary (lower) section, the mean loosening torque values for the fully retrievable groups were 39.34 Ncm for BC1.0 and 37.31 Ncm for KC1.0. The percentage torque loss for the fully retrievable groups was 18.32% for BC1.0 and 17.34% for KC1.0.

### 3.1. Tightening Torque vs. Loosening-to-Tightening Ratio (%)

Neither prototype exhibited a statistically significant correlation between peak tightening torque and the loosening-to-tightening ratio. For BC1.0, Pearson’s coefficient was r = 0.135 (*p* = 0.632) and Spearman’s coefficient was ρ = −0.032 (*p* = 0.909). For KC1.0, Pearson’s coefficient was r = 0.364 (*p* = 0.182) and Spearman’s coefficient was ρ = 0.443 (*p* = 0.098). Although the Klockner system showed a moderate positive tendency, neither coefficient reached the threshold for statistical significance.

### 3.2. Tightening Torque vs. Loosening Torque

BC1.0 demonstrated a strong, statistically significant positive correlation between peak tightening torque and peak loosening torque: Pearson’s coefficient was r = 0.882 (*p* < 0.001), and Spearman’s coefficient was ρ = 0.878 (*p* < 0.001). The coefficient of determination (R^2^ ≈ 0.78) indicates that approximately 78% of the variability in loosening torque can be explained by tightening torque. Specimens tightened with greater torque consistently required greater torque for loosening.

KC1.0 exhibited a weaker relationship. Pearson’s coefficient was moderate and statistically significant (r = 0.591, *p* = 0.020), whereas Spearman’s rank correlation was notably lower and non-significant (ρ = 0.261, *p* = 0.348). The R^2^ value of approximately 0.35 indicates that only 35% of the variability in loosening torque was attributable to tightening torque.

## 4. Discussion

The mechanical reliability of screw-retained implant restorations depends not only on adequate preload generation and maintenance but also on the clinician’s ability to manage mechanical complications over time. This article aimed to present a modified abutment screw design, test it, and characterise its torque behaviour during tightening and loosening. The present findings suggest that novel abutment screw head designs incorporating a retrievability feature, such as the one presented in this article, represent a relevant development, as they address both biomechanical performance and long-term clinical manageability.

This study used the Brånemark 4.1 and Klockner VEGA RV systems. The rounded lobe geometry increases the driver–screw contact surface area, thereby distributing applied forces more evenly and conferring greater resistance to plastic deformation compared with conventional hexagonal designs [9,15,18]. The negative flank angle of approximately 15° maintains driver engagement under elevated torque loads, effectively preventing cam-out during tightening procedures [9,16]. However, when deformation occurs, the lobes fail uniformly and progressively, leaving no residual angular geometry for conventional retrieval instruments to engage, thereby creating a paradox that renders extraction considerably more challenging than in stripped hexagonal screws.

The proposed screw head design incorporates a secondary engagement geometry intended to remain functional even after partial deformation or surface wear of the primary torque interface, thereby reducing procedure complexity, chair time, and the risk of damage to the implant’s internal connection [5,9,11,25]. Initially, the concept was to create a secondary geometry compatible with a universally available screwdriver and with a similar stress distribution along the screw head walls, without increasing screw head height by more than 1 mm [35]. The first design had a 0.9 mm hexagon. In Brånemark-compatible screws, there was no need to increase screw head height. In contrast, in the Klockner-compatible groups, initial tests performed by the research group revealed difficulty in engaging the screwdriver within the secondary geometry, and wear occurred at the screwdriver angles during screw retrieval attempts. During experimental research, it was hypothesised that, in the Klockner system, there was a minimal tolerance between the screwdriver and the 0.9 mm hexagon because the 0.05 mm discrepancy corresponds to approximately 5% of the interface dimension, which appears to be critical in this context. From a biomechanical perspective, increasing the effective contact area from 0.9 mm to 1.0 mm and reducing sharp internal angles promotes a more uniform stress distribution during torque application because of the approximately 11% increase in contact area, thereby decreasing peak contact stresses and plastic deformation [9,17,18]. Based on these preliminary outcomes, the design evolved to incorporate a 1.0 mm hexagon for both types of screw. Consequently, both Brånemark- and Klockner-compatible screws were manufactured with increased head height to preserve adequate wall thickness and mechanical resistance [9,18]. These design principles are consistent with established concepts governing preload generation and mechanical stability in implant abutment screws [4,6,24,37,38,39].

From a statistical point of view, the differences between prototypes were more pronounced than expected, and in at least two of the four groups, the implications for clinical practice are difficult to overlook. One of the most relevant findings was the failure to retrieve screws in most specimens from BC0.9 and KC0.9, where the retrieval screwdriver and head geometry incorporated a 0.9 mm hexagon with a 1 mm height. This was not an isolated occurrence or a testing artifact; it was consistently observed throughout the testing of the fifteen screws in each group, indicating a fundamental design limitation. When a screw cannot be reliably removed under controlled laboratory conditions, using calibrated instruments and without biological tissue interference, it is reasonable to question how it would perform in a clinical scenario in which access is restricted, visibility is limited, and the consequences of failure are considerably more serious. The literature on stripped and fractured prosthetic screws is clear: once socket geometry is compromised, clinical options rapidly diminish [5,11,22], and what begins as a prosthetic complication can escalate into a surgical problem [10,11,27].

BC1.0 achieved the highest mean peak tightening torque among the four groups, at 48.17 Ncm, followed by KC1.0 at 45.56 Ncm. The difference between the two systems is modest in absolute terms. Still, it may reflect underlying differences in applied torque, thread pitch, surface treatment, or driver–screw contact area—all factors known to influence the efficiency of torque-to-preload conversion and the degree of mechanical deformation under repeated tightening [16,24,38,39].

The two retrievable prototypes showed a similar pattern of torque loss during loosening: a proportional loss of 18.32% for BC1.0 and 17.34% for KC1.0. This convergence is noteworthy. Despite differences in screw head geometry and absolute torque values, the two systems appear to lose a nearly identical proportion of their initial preload upon removal, suggesting that torque retention efficiency may be more closely related to implant–abutment interface characteristics than to specific driver design. This observation is broadly consistent with previous studies on preload relaxation and embedding loss in titanium implant components [3,21], which recognized interface settlement and elastic recovery as the primary mechanisms responsible for preload reduction rather than the tightening force magnitude alone [4,24,37,40].

The correlation analysis between peak tightening torque and the loosening-to-tightening ratio yielded no statistically significant result in either prototype. These findings indicate that the magnitude of the applied tightening torque does not reliably predict the proportion of torque retained upon loosening in either system, which challenges the intuitive clinical assumption that reducing tightening torque might facilitate retrieval. Interestingly, the retrieval ratio appears to be primarily governed by interface-level mechanics rather than by the magnitude of the applied force, with practical implications for screw selection and tightening protocols [3,4,24,37,38,39,40].

The correlation analysis between peak tightening torque and peak loosening torque revealed a more differentiated pattern. In BC1.0, the relationship was strong and consistent and was confirmed by both parametric and nonparametric approaches. In practical terms, this indicates that, in the Brånemark-compatible prototype, greater tightening torque reliably translated into greater loosening torque, reflecting mechanically coherent and predictable behaviour [4,38,40]. The close agreement between Pearson and Spearman coefficients further supports the robustness of this relationship and suggests that it was not driven by outlier specimens. From a clinical design standpoint, this level of mechanical consistency is highly desirable in a retrievable screw system [11,24,25]. KC1.0 presented a more ambiguous pattern: Pearson’s test demonstrated a moderate and statistically significant correlation, whereas Spearman’s rank coefficient was substantially lower and failed to reach statistical significance. This divergence suggests that the linear association detected by Pearson’s test may have been driven by a small number of extreme observations rather than by a consistent monotonic trend. One plausible contributor to this discrepancy is the driver replacement that occurred during KC1.0 testing.

When both screw systems were compared, BC1.0 demonstrated stronger, more robust correlations than KC1.0 across both analyses. Whereas both parametric and nonparametric tests confirmed the tightening-to-loosening relationship in BC1.0, the KC1.0 system yielded a significant result only with Pearson’s test, a finding not corroborated by Spearman’s rank-based analysis. This discrepancy may reflect greater mechanical variability at the KC1.0 screw–implant interface or the influence of confounding factors, different implant connections, or the hex driver replacement required during testing of the KC1.0 specimens. One possible explanation lies in the geometry of the Klockner screw head: the octagonal star design, with its eight rounded lobes, offers excellent resistance to cam-out under normal clinical conditions [9,15,16,18], but the same geometry may provide less reproducible torque transmission when driver wear or minor misalignment reduces the effective contact area [16,38,39]. In the present study, this may have been accentuated by the progressive wear of the retrieval driver across the fifteen KC1.0 specimens, which necessitated its replacement during testing and could have introduced variability in torque transmission between specimens.

The present study has several limitations that should be considered when interpreting the findings. As an in vitro investigation, the experimental conditions do not fully reproduce the clinical environment, where saliva, restricted access, cyclic occlusal loading, contamination, and prosthetic misfit may influence screw behaviour and retrievability. Moreover, the sample size was limited, and only two implant systems were evaluated, which may restrict the generalizability of the results to other implant–abutment connections. In addition, with n = 15 the study was adequately powered only for strong correlations (r ≥ 0.67); weaker associations may therefore have gone undetected, and the non-significant correlations should be regarded as inconclusive rather than as evidence of no relationship. No unmodified commercial screw was included as a control group, since a conventional screw lacks a secondary interface and cannot serve as a comparator for the retrieval outcome; consequently, the absolute torque values reported here should be interpreted with caution. A direct comparison between the 1.0 mm design and commercially available screws was carried out by our group in a separate finite element study. In addition, deformation of the primary interface was not experimentally induced: retrieval was tested exclusively through the secondary interface, simulating the situation in which the primary interface is no longer usable. Experimentally inducing standardised primary-head deformation and then testing retrieval through the secondary interface represents an important next step. In addition, long-term mechanical performance was not assessed because cyclic fatigue testing, preload stability evaluation, fracture resistance analysis, and finite element simulations were not performed. Despite adding 1 mm, the increased screw head height required to accommodate the secondary retrieval geometry may not represent a limitation in situations with reduced restorative space. Furthermore, the incorporation of a secondary internal chamber modifies the cross-sectional geometry and local stress distribution of the screw head, which could influence its strength, fracture resistance, and long-term stability; none of these properties were evaluated in the present study. The added head height also entails a geometric trade-off that may be relevant when vertical restorative space is limited, and the manufacturing feasibility of the secondary interface at these dimensions, although demonstrated here at the prototype level, would require confirmation for large-scale production. These aspects, including fatigue behaviour and comparison with commercial screws, have been examined in two complementary studies conducted by our group with 1.0 mm screws. Future studies should therefore include dynamic fatigue protocols and long-term clinical investigations to validate the mechanical reliability and clinical applicability of the proposed design.

Within the limitations inherent to in vitro mechanical evaluation, the present design approach suggests that integrating a retrievability feature into screw head geometry may represent a clinically useful approach rather than an auxiliary modification. These findings should be interpreted as preliminary laboratory evidence rather than as clinical validation. Within these constraints, the consistent retrievability observed in BC1.0 and KC1.0, combined with their acceptable torque loss profiles, suggests that both prototypes may warrant further evaluation as retrievable prosthetic screw systems, a meaningful outcome given that retrievability should be considered a prerequisite for any design intended for routine clinical use. Loss of screw retrievability following deformation or fracture is a well-recognized complication that may necessitate invasive retrieval procedures or, in challenging situations, implant removal.

## 5. Conclusions

The present article introduces a new prosthetic screw head design featuring two distinct screwdriver engagement sections. This feature may have clinical relevance as a simple retrieval system. When the use of the original screwdriver in the primary interface is not possible due to deformation or damage to the primary geometry, the secondary lower section, featuring a 1.0 mm hexagonal geometry, demonstrated 100% retrieval success under the in vitro conditions tested for loosening in both Brånemark- and Klockner-compatible screws. These findings should be regarded as a proof-of-concept obtained under laboratory conditions and require further mechanical and clinical investigation, including fatigue and cyclic loading testing, evaluation across additional implant systems, and comparison with commercial screws, before clinical application can be considered.

## 6. Patents

The authors declare that University of Porto filed a patent application for the design described in this manuscript prior to publication (application No. 121805; provisional title: «Abutment Screw Head Design with an Integrated Retrieval Feature»; filing date: 23 August 2026).

## Figures and Tables

**Table 1:** Summary of correlation results.

Prototype	Pair of Variables	Pearson r	*p*-Value	Spearman ρ	*p*-Value	Significant
BC1.0	Tightening vs. % Loosening/Tightening	0.135	0.632	−0.032	0.909	No
BC1.0	Tightening vs. Loosening	0.882	<0.001	0.878	<0.001	Yes
KC1.0	Tightening vs. % Loosening/Tightening	0.364	0.182	0.443	0.098	No
KC1.0	Tightening vs. Loosening	0.591	0.020	0.261	0.348	Pearson only

## Data Availability

The data presented in this study are available on request from the corresponding author due to legal reasons.

## Abbreviations

The following abbreviations are used in this manuscript:

BC	Brånemark-compatible
KC	Klockner-compatible

## Appendix A

**Table A1:** Raw data from testing methodology—retrievability test.

**Group—Screw**	**Tightening (Ncm)** **Hex 1.2 mm**	**Loosening (Ncm)** **Hex 0.9 mm**	**% Torque Loss**
BC0.9—screw 1	39.829	33.310	16.37%
BC0.9—screw 2	37.754	33.476	11.33%
BC0.9—screw 3 to screw 15	±38	Failed to unscrew	-
**Group—Screw**	**Tightening (Ncm)** **Hex 1.2 mm**	**Loosening (Ncm)** **Hex 1.0 mm**	**% Torque Loss**
BC1.0—screw 1	45.017	36.338	19.28%
BC1.0—screw 2	50.993	42.309	17.03%
BC1.0—screw 3	46.179	37.001	19.87%
BC1.0—screw 4	49.043	38.494	21.51%
BC1.0—screw 5	45.930	37.623	18.09%
BC1.0—screw 6	48.088	41.355	14.00%
BC1.0—screw 7	46.262	39.448	14.73%
BC1.0—screw 8	47.880	39.904	16.66%
BC1.0—screw 9	46.054	36.006	21.82%
BC1.0—screw 10	54.521	43.844	19.58%
BC1.0—screw 11	46.801	39.157	16.33%
BC1.0—screw 12	48.669	40.194	17.41%
BC1.0—screw 13	48.545	40.319	16.95%
BC1.0—screw 14	53.442	42.097	21.23%
BC1.0—screw 15	45.141	36.006	20.24%
**Group—Screw**	**Tightening (Ncm)** **Hex 1.2 mm**	**Loosening (Ncm)** **Hex 0.9 mm**	**% Torque Loss**
KC0.9—screw 1	38.860	36.003	7.35%
KC0.9—screw 2	38.210	30.577	19.98%
KC0.9—screw 3	39.995		
KC0.9—screw 4	37.837	30.573	19.20%
KC0.9—screw 5	39.206		
KC0.9—screw 6	38.127	32.682	14.28%
KC0.9—screw 7	38.293	31.776	17.02%
KC0.9—screw 8	37.795	31.154	17.57%
KC0.9—screw 9	39.481		
KC0.9—screw 10	37.961	29.910	21.21%
KC0.9—screw 11	39.621		
KC0.9—screw 12	37.463	31.710	15.36%
KC0.9—screw 13	37.122	30.905	16.75%
KC0.9—screw 14	38.501		
KC0.9—screw 15	38.677	32.173	16.82%
**Group—Screw**	**Tightening (Ncm)** **Hex 1.2 mm**	**Loosening (Ncm)** **Hex 1.0 mm**	**% Torque Loss**
KC1.0—screw 1	43.000	35.300	17.91%
KC1.0—screw 2	40.700	32.149	21.01%
KC1.0—screw 3	44.350	33.400	24.69%
KC1.0—screw 4	43.233	34.456	20.30%
KC1.0—screw 5	47.797	41.521	13.13%
KC1.0—screw 6	47.800	32.771	31.44%
KC1.0—screw 7	45.515	39.075	14.15%
KC1.0—screw 8	57.841	38.909	32.73%
KC1.0—screw 9	56.845	51.800	8.88%
KC1.0—screw 10	41.960	41.646	0.75%
KC1.0—screw 11	46.900	37.125	20.84%
KC1.0—screw 12	39.497	35.964	8.94%
KC1.0—screw 13	41.320	34.305	16.98%
KC1.0—screw 14	40.451	37.291	7.81%
KC1.0—screw 15	46.280	34.015	26.50%
